# First Insight into the Neuroprotective and Antibacterial Effects of Phlorotannins Isolated from the Cell Walls of Brown Algae *Fucus vesiculosus* and *Pelvetia canaliculata*

**DOI:** 10.3390/antiox12030696

**Published:** 2023-03-11

**Authors:** Darya Meshalkina, Elena Tsvetkova, Anastasia Orlova, Renata Islamova, Maria Grashina, Daria Gorbach, Vladimir Babakov, Antonio Francioso, Claudia Birkemeyer, Luciana Mosca, Elena Tarakhovskaya, Andrej Frolov

**Affiliations:** 1 Faculty of Biology, St. Petersburg State University, 199034 St. Petersburg, Russia; 2 Sechenov Institute of Evolutional Physiology and Biochemistry, 194223 St. Petersburg, Russia; 3 Department of General Pathology and Pathological Physiology, Institute of Experimental Medicine, 197022 St. Petersburg, Russia; 4 Laboratory of Analytical Biochemistry and Biotechnology, K.A. Timiryazev Institute of Plant Physiology RAS, 127276 Moscow, Russia; 5 Research Institute of Hygiene, Occupational Pathology and Human Ecology, Federal Medicobiological Agency, 188663 St. Petersburg, Russia; 6 Department of Biochemical Sciences, Sapienza University, 00185 Roma, Italy; 7 Department of Bioscience and Technology for Food, Agriculture and Environment University of Teramo, 64100 Teramo, Italy; 8 Faculty of Chemistry and Mineralogy, University of Leipzig, 04103 Leipzig, Germany; 9 German Center for Integrative Biodiversity Research (iDiv), 04103 Leipzig, Germany; 10 Vavilov Institute of General Genetics RAS, St. Petersburg Branch, 190000 St. Petersburg, Russia

**Keywords:** Alzheimer’s disease, antibacterial, antioxidant, anti-neurodegenerative, brown algae, cell culture, cell wall-bound phlorotannins, neuroprotective, Parkinson’s disease, Phaeophyceae, phlorotannins

## Abstract

Phaeophyceae (brown algae) essentially contribute to biotopes of cold and temperate seas. Their thalli are rich in biologically active natural products, which are strongly and universally dominated with phlorotannins—polyphenols of complex and diverse structure based on multiple differently arranged phloroglucinol units and well known as strong antioxidants with a broad spectrum of biological activities. In the algal cells, phlorotannins can either accumulate in the cytoplasm or can be secreted into the cell wall (CW). The biological activities of extractable intracellular phlorotannins have been comprehensively characterized, whereas the properties of the CW-bound polyphenol fraction are still mostly unknown. Recently, we identified dibenzodioxin bonding as the principal structural feature of the CW-bound phlorotannins in fucoid algae, whereas soluble intracellular phlorotannins rely on aryl and ether bonds. However, profiles of biological activity associated with these structural differences are still unknown. Therefore, to the best of our knowledge, for the first time we address the antioxidant, cytotoxic, neuroprotective, and antibacterial properties of the CW-bound phlorotannin fractions isolated from two representatives of the order Fucales—*Fucus vesiculosus* and *Pelvetia canaliculata*. The CW-bound phlorotannins appeared to be softer antioxidants, stronger antibacterial agents and were featured with essentially less cytotoxicity in comparison to the intracellular fraction. However, the neuroprotective effects of both sub-cellular phlorotannin fractions of *F. vesiculosus* and *P. canaliculata* were similar. Thus, due to their lower cytotoxicity, CW-bound phlorotannins can be considered as promising antioxidants and neuroprotectors.

## 1. Introduction

Brown algae comprise 256 genera with 2040 species, 95% of which inhabit cold and temperate waters [1]. About 90 species of brown algae, rich in biologically active metabolites, can be found in the global coastline area, which makes them commercially important organisms [2]. Phlorotannins represent the most abundant group of valuable secondary metabolites (depending on species, season and habitat, accounting for 0.5 to 25% of dry weight (DW) [3]), which are unique to brown algae and are present at detectable concentrations across almost all orders of Phaeophyceae [4]. Representatives of the family Fucaceae, such as *Fucus vesiculosus* and *Pelvetia canaliculata*, are among the most phlorotannin-rich species and contain from 5 to 17% DW of phlorotannins, depending on the thallus zone [5]. These polyphenols act as integral components of cell walls (CW), antioxidants, metal chelators and herbivore protectants [4,6].

**Figure 1:**
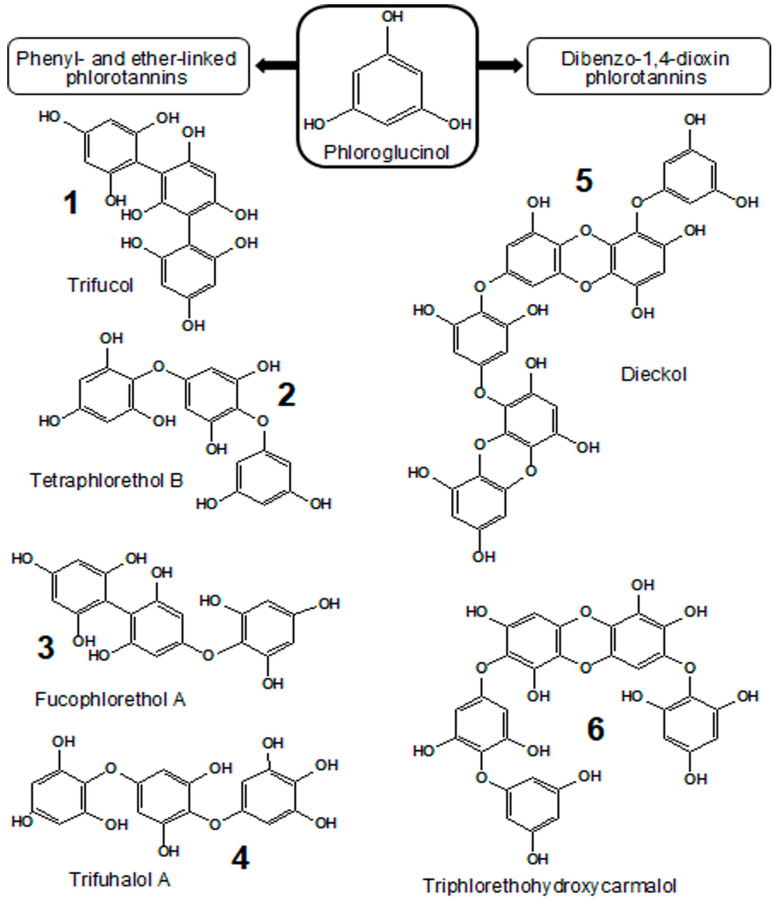
Structures of phloroglucinol and representatives of major phlorotannin classes: fucols (1), phlorethols (2), fucophlorethols (3), fuhalols (4), eckols (5), and carmalols (6) [11].

Natural phlorotannins represent complex mixtures of water-soluble oligo- and polymerization products of phloroglucinol (1,3,5-trihydroxybenzene, Figure 1), with molecular sizes ranging from 126 Da (monomer molecule) to 650 kDa [7]. Based on their structure (i.e., the types of inter-monomer chemical bonds), these compounds can be split into four classes: (i) fuhalols and phlorethols with ether linkages (aryl-*O*-aryl); (ii) phenyl-linked fucols (aryl-aryl); (iii) fucophlorethols, which possess both ether and phenyl linkages and can have a branched structure; and (iv) dibenzodioxin-linked eckols and carmalols [8]. The high isomerization of phlorotannins results in an impressive structural diversity of these phenolic compounds [9]. Thus, the distinct molecular size fractions (<1, 1–10, 10–100, and >100 kDa) of phlorotannins demonstrate high variability, depending on the species, geographical region, and thallus zone [10]. This fact and the absence of commercially available standards make the characterization of phlorotannin profiles a challenging problem [11], which in the most efficient way can be addressed by ultra-performance liquid chromatography-tandem mass spectrometry (UHPLC-MS/MS) and nuclear magnetic resonance (NMR) techniques [12,13].

Algal cells usually contain a pool of intracellular phlorotannins in the specialized organelles, physodes, which are likely formed in the endoplasmic reticulum (ER) and Golgi apparatus. Phlorotannin precursors might be synthesized in the ER and then transferred to the Golgi for further processing [14]. Histochemical studies distinguish at least two types of physodes: the vesicles accumulating around the nucleus and those moving to the periphery of the cytoplasm and then secreting their contents into the apoplast, where phlorotannins form complexes with alginic acid [15]. The content of CW-bound phlorotannins in the algal tissue is an order of magnitude lower than the concentration of soluble ones [16]. Thus, blades of *F. vesiculosus* contain 16% DW of intracellular phlorotannins and 0.8% DW of CW-bound phlorotannins, and blades of *P. canaliculata* contain 8 and 0.4% DW, respectively [5]. At present, the fraction of phlorotannins associated with the cell wall is almost unstudied.

Recently, due to their biological activity, phlorotannins have attracted special attention by food chemists, cosmetics producers and pharmacists [16]. Similar to the polyphenols of vascular plants, phlorotannins exhibit pronounced antioxidant properties [17], which are clearly manifested with high antioxidant capacity and reactive oxygen species (ROS) scavenging activity comparable to those of ascorbic acid, butylated hydroxytoluene (BHT) and propyl gallate [18,19]. Moreover, phlorotannin-rich algal extracts were shown to suppress lipid peroxidation and formation of hydroperoxides with an efficiency comparable to that of propyl gallate [20].

Moreover, phlorotannins exhibit pronounced antimicrobial effects towards both Gram-positive and -negative microorganisms, which were comprehensively reviewed by Catarino et al. [16]. In addition, phlorotannins were proven to be anti-inflammatory [21,22,23], cytotoxic and antiproliferative compounds [24,25,26]. Phlorotannin preparations effectively inhibited α-amylase and α-glucosidase activities, i.e., they exerted anti-diabetic effect [27,28]. Both phloroglucinol and low-molecular-weight phlorotannins reduced formation of advanced glycation end-products (AGEs), which are responsible for such diabetic complications as retinopathy, neuropathy and cardiomyopathy [29].

Unfortunately, although the biological activity of soluble phlorotannins is well characterized, the properties of the cell wall polyphenolic fraction are still insufficiently addressed and remain mostly unknown. Recently, in a comprehensive high resolution–mass spectrometry (HR-MS) survey [5], we unambiguously showed that the CW-bound phlorotannin fraction of three fucoid algae (including *F. vesiculosus* and *P. canaliculata*) is clearly differentiated by its structure (i.e., monomer unit/bonding type and degree of polymerization) from the soluble one (aryl/ether vs. dibenzodioxin-type units, Figure S1). As this structural diversity might underlie differential biological activity, here we address the antioxidant, antimicrobial and neuroprotective (antineurodegenerative) effects of the cell wall fractions isolated from *F. vesiculosus* and *P. canaliculata* thalli and compare their properties with their soluble counterparts.

## 2. Materials and Methods

### 2.1. Algal Material

Samples of two species of Fucales (*Fucus vesiculosus* L. and *Pelvetia canaliculata* (L.) Dcne and Thur.) were collected in the Keret Archipelago (Kandalaksha Bay, White Sea; 66°17′28.76′′ N, 33°40′03.46′′ E) in August 2020. Mature thalli from the typical habitats of each species (low-intertidal and high-intertidal zones, respectively) were frozen and transported to the laboratory for subsequent homogenization and phlorotannin extraction.

### 2.2. Materials

Unless stated otherwise, materials were obtained from the following manufacturers: Duchefa Biochemie (Haarlem, The Netherlands): dimethylsulfoxide (>99.9 atom % D); Ecos-1 (Moscow, Russia): *n*-hexane (analytical grade), ethyl acetate (analytical grade), methylene chloride (analytical grade); Honeywell (Seelze, Germany): acetonitrile (>99.9%, LC-MS grade). All other chemicals were purchased from Merck KGaA (Darmstadt, Germany). Water was purified in house with a water conditioning and purification system, the GenPure Pro UV-TOC system (resistance 18 mΩ/cm, Thermo Fisher Scientific, Langenselbold, Germany).

### 2.3. Extraction of the Intracellular and CW-Bound Phlorotannins from the Algal Material

Extraction of intracellular and CW-bound phlorotannins was performed according to [14] with modifications. Briefly, samples of 1–2 g frozen algal material were homogenized using a cryogenic laboratory mill Freezer/Mill 6870 (SPEX SamplePrep, Germany), transferred to the 15 mL falcon tubes, poured with 10 mL of acetone:water (70:30, *v*/*v*) and left soaking for one hour. Afterwards, each extract was centrifuged (5000× *g*, 10 min), the supernatant was transferred to a fresh tube, and the pellet was re-extracted with another 10 mL of aqueous acetone. The supernatants of five extraction rounds were combined, and acetone was evaporated under reduced pressure in a centrifugal evaporator (CentriVap vacuum concentrator system, Labconco, Kansas City, Mo, USA). The CW-bound phlorotannin fraction was extracted from the precipitate of the remaining algal material after the extraction of intracellular phlorotannins. First, the precipitate was washed three further times (i.e., re-suspended each time in 10 mL of aqueous acetone and centrifuged at 5000× *g* for 10 min with supernatants discarded) to remove the possible remnants of the intracellular phenolics. Then, the precipitate was resuspended in 5 mL of 1 mol/L aqueous NaOH solution (80 °C) and then incubated for 2.5 h at room temperature (RT) with continuous shaking (750 rpm). After centrifugation (5000× *g*, 10 min) the supernatant was transferred to another tube. The alkaline extraction was repeated three times. The combined supernatants were neutralized with concentrated HCl to pH 6.8–7.0. At the next step, both intracellular and CW-bound phlorotannin extracts were defatted three times, partitioning against dichloromethane (1:1, *v*/*v*), and phlorotannins were extracted by five successive portions of ethyl acetate (1:1, *v*/*v*). Ethyl acetate extracts were dried and resuspended in 1 mL water.

The detailed chemical characterization of these semi-purified extracts was done in our previous study: using HPLC-MS analysis, we confirmed that phlorotannins were the major constituents of the extracts and revealed the specific molecular profiles of both intracellular and CW-bound phlorotannin extracts of *F. vesculosus* and *P. canaliculata* [5].

A modification of the Folin–Ciocalteu micro-method was used to measure the phlorotannin content in the intracellular and CW-bound phlorotannin extracts [30]. Phloroglucinol (Sigma-Aldrich 79330) was used as the standard. The reaction mixture containing 0.3 mL of sample (diluted as necessary), 0.3 mL of Folin reagent and 2.4 mL of 5% (*w*/*v*) aqueous Na_2_CO_3_ was incubated for 20 min at 45 °C, and then absorbance was measured at 750 nm using a SPEKOL 1300 spectrophotometer (Analytik Jena AG, Jena, Germany).

### 2.4. Antioxidant Assays

The antioxidant effects of the extracts enriched in intracellular and CW-bound phlorotannins isolated from the thalli of *F. vesiculosus* and *P. canaliculata* were addressed by 2,2-diphenyl-1-picrylhydrazyl (DPPH) free radical scavenging, Trolox equivalent antioxidant capacity (TEAC) and nitroblue tetrazolium (NBT) assays. The analyses were accomplished according to Masci et al. [31], with minor modifications as follows.

#### 2.4.1. DPPH Free Radical Scavenging Effect

The phlorotannin-rich extracts were diluted with distilled water to achieve the concentration of 1 mg/mL. The 10 µL aliquots of these solutions (10 µg in total) were supplemented to 1 mL portions of 40 μmol/L methanolic solution of stable nitrogen-centered free radical DPPH•. The absorbance was monitored spectrophotometrically at 517 nm after 1 h of incubation at RT. The capacity of the phlorotannin-rich preparations for scavenging of the DPPH• radical was estimated from the difference in the absorbance acquired in the presence and absence of the algal isolates. The corresponding values were expressed as the percentage of DPPH• consumption as a function of the sample concentration. Thereby, the oxidant activities of the algal extracts were determined as relative values normalized to the antioxidant activity of ascorbic acid (taken as 100%).

#### 2.4.2. Trolox Equivalent Antioxidant Capacity (TEAC) Assay

The 2,2′-azinobis(3-ethylbenzothiazoline-6-sulfonic acid) diammonium salt (ABTS) was dissolved in water to obtain a 7 mmol/L solution, which was further oxidized to corresponding radical cation (ABTS+) in the presence of 2.45 mmol/L potassium persulfate (16 h at RT in dark). The radical cation reagent (ABTS+) was diluted with ethanol to obtain the absorbance of 0.70 (±0.02) at 734 nm. Aliquots of the phlorotannin samples, diluted as described in the previous section, were supplemented with 1 mL of the ABTS+ solution. Absorbance was measured at 734 nm after six minutes of incubation in the dark at RT. Antioxidant capacities of the phlorotannin-rich extracts were reported as equivalents of Trolox.

#### 2.4.3. Assessment of Extract Capacity to Scavenge Superoxide Anion Radicals (NBT Assay)

The stock solutions (1 mmol/L) of phenazine methosulfate (PM) in ethanol, NBT in water, and β-NADH in 0.05 mol/L phosphate buffer (pH 7.4) were freshly prepared daily. The reaction mixtures contained 73 μmol/L β-NADH, 15 μmol/L PM, 50 μmol/L NBT, and 10 μg of samples in 1 mL of 0.02 mol/L Tris-HCl buffer, pH 8.0. The absorbance was determined at 560 nm immediately after mixing the reagents and after 15 s of reaction. The change of absorbance in time (ΔAbs/min) and absorption coefficient of 1 μmol/L formazan solution 0.03 were used to calculate the rate of production of superoxide anion radicals.

### 2.5. Antimicrobial Assays

The analysis of the antimicrobial activity was accomplished as described by Orlova et al. [32] with changes. Thereby, the minimum inhibitory concentrations (MIC) of the intracellular and CW-bound phlorotannin extracts were determined by the broth microdilution method, as recommended by the Clinical Laboratory Standards Institute, USA [33]. The following bacterial strains were cultured under aerobic conditions according to the approved standard protocol: *Escherichia coli* ATCC 25922, *Pseudomonas aeruginosa* ATCC 27853, *Listeria monocytogenes* EGD, *Staphylococcus aureus* ATCC 25923, *Staphylococcus aureus* SG-511, MRSA ATCC 33591, *Micrococcus luteus* CIP A270. Strains MRSA ATCC 33591 and *Listeria monocytogenes* EGD were provided by Prof. R. Lehrer (University of California Los Angeles, CA, USA); *Escherichia coli* ATCC 25922, *Pseudomonas aeruginosa* ATCC 27853, *Micrococcus luteus* CIP A270, *Staphylococcus aureus* ATCC 25923—Department of Molecular Microbiology, IEM; strain *Staphylococcus aureus* SG 511—by Professor H.G. Sahl (University of Bonn, Bonn, Germany). The microorganisms from an agar plate culture were incubated for 2–6 h in 2.1% (*w*/*v*) Mueller-Hinton broth (MHB, HiMedia, Mumbai, India) at 37 °C on an orbital shaker at an agitation rate 150 rpm. After adjusting the turbidity to 0.5 McFarland (1.5 × 10^8^ CFU/mL), suspensions were diluted in sterile 2.1% (*w*/*v*) MHB until achieving a final bacterial concentration of 1.0 × 10^6^ CFU /mL.

The intracellular and CW-bound phlorotannin extracts from *F. vesiculosus* and *P. canaliculata* were serially two-fold diluted (the initial concentration was 500 μg/mL for intracellular phlorotannins and 125 μg/mL for CW-bound phlorotannins) with sterile 2.1% (*w*/*v*) MHB, and 50 μL aliquots were added to the wells of a 96-well sterile U-shaped plate (GreinerBio-one, Austria). Afterwards, 50 μL of bacterial suspension was added to each well. The controls of bacterial growth and viability and medium sterility were included. The microtiter plates were incubated aerobically without shaking at 37 °C for 18 h. MICs were defined as the lowest extract concentrations that inhibited the visual growth of microorganisms. The experiments were performed in triplicate, and the final results were calculated as the medians based on the data from three independent experiments, each accompanied with the complete set of the controls.

### 2.6. Assessment of Antineurodegenerative Effects

Evaluation of the anti-neurodegenerative effects relied on the cell models of Alzheimer’s and Parkinson’s diseases established with SH-SY5Y human neuroblastoma cells and amyloid peptide Aβ25–35

#### 2.6.1. Cell Culture

Human neuroblastoma SH-SY5Y cells were obtained from ICLC (Genova, Italy). Cells were cultured in T25 cell culture flasks (Eppendorf, Germany) in DMEM/F12 with 10% fetal bovine serum (FBS), 2 mmol/L L-glutamine in the atmosphere of 95% air and 5% CO_2_ at 37 °C and humidity ≥95%. Cells were passed twice per week by detaching with accutase (2–3 min followed with dilution with full medium). Before the accutase treatment, the cells were washed two times with 1 mL of PBS, pH 7.4. The cells were not split in ratios lower than 1:3–1:5. Cell counting relied on an automatic cell counter (TC20, Bio Rad, CA, USA) or manual calculation with a Neubauer chamber.

#### 2.6.2. Synthesis and Aggregation of Aβ25–35 Amyloid Peptide

Aβ25–35 was obtained by solid phase peptide synthesis as described elsewhere [26] and stored at −20 °C. The peptide was resuspended in 1,1,1,3,3,3-hexafluoro-2-propanol, incubated for 1 h with gentle shaking at 4 °C, then aliquoted, and the solvent was completely evacuated under reduced pressure by centrifugal evaporation in SpeedVac during 20 min. The aliquots were stored under a vacuum glass bell. The day before being used, aliquots were re-suspended in phosphate buffered saline (PBS) at a final concentration of 1 mmol/L and incubated in an ultrasonic bath on ice for 30 min to induce aggregation, followed by gentle shaking at 4 °C overnight. Directly prior to cell treatment, Aβ_25–35_ was diluted in culture medium at the final concentration of 30 µmol/L.

#### 2.6.3. Assay of Neuroprotection in the Model of Alzheimer’s Disease–Related Toxicity

Cells were seeded at the density of 4 × 10^4^ cells per each well of 96-well cell culture treated microtiter plates (Eppendorf, Germany). The next morning, the medium was discarded, and 50 µL of fresh medium supplemented with serially diluted phlorotannin-rich extracts (1–10 µg/mL, n = 4) was added to each well. Half an hour later, 50 µL of aggregated Aβ25–35 peptide was added to each well to yield the concentration of 25 μmol/L. The control cells were supplemented with 50 μL of fresh medium. After incubation for 24 h at 37 °C, the methylthiazolyldiphenyl-tetrazolium bromide (MTT) test was accomplished in quadruplicate. For this, 5 mg/mL MTT solution in PBS (pH 7.4) was added to each well to obtain the final concentration in culture medium of 0.5 mg/mL. After a 2 h incubation at 37 °C in a CO_2_ incubator (Sanyo, Kyoto, Japan), the medium was removed, and formazan crystals were dissolved in 100 µL/well of DMSO. Absorbance was measured by DU-50 (Beckman Coulter, Indianapolis, IN, USA) at 570 nm with a reference at 630 nm. Cell viability was calculated for each well as a ratio of specific absorbance to mean absorbance obtained for untreated cells (with blanks subtracted).

#### 2.6.4. Assay of Neuroprotection in the Model of Parkinson’s Disease–Related Toxicity

For the assessment of the anti-Parkinsonian activity of the phlorotannin extracts, we used the paraquat model of neurodegeneration established on differentiated SH-SY5Y cells. The day before the differentiation procedure, the cells were seeded into the wells of a 96-well plate at the concentration 50,000 cells per well. SH-SY5Y cell differentiation was induced by culture growth in DMEM/F-12 medium with 3% FBS containing 10 μmol/L retinoic acid and 10 μmol/L phorbol myristate acetate. Differentiation proceeded for 10 days, with medium exchange every two days, and was routinely controlled visually by phase contrast microscopy. At the day of experiment, the algal extracts were added to the cells for 30 min in 50 μL of culture medium (1–10 μg/mL). Neurodegeneration was modeled by the toxicity of 800 μmol/L paraquat incubated for 24 h with differentiated cells, resulting in 50% viability of the culture. Afterwards, MTT solution was applied, and the viability values were retrieved as described in Section 2.6.3 for anti-Alzheimer’s assay.

### 2.7. Statistical Analysis

The contents of the individual compounds as well as antioxidant and anti-neurodegenerative activities were expressed as the mean (mean ± standard deviation). Statistical significance of inter-group differences was assessed by the Mann–Whitney test (*p* ≤ 0.05) with Bonferroni correction for multiple comparisons applied at the confidence level of *p* ≤ 0.05.

## 3. Results

For all analyses, extract concentrations were normalized to the total phenolic contents. Phlorotannins highly dominate in the phenolic profiles of brown algae (especially fucoids), and it was shown that non-phenolic compounds contribute less than 5% of Folin–Ciocalteu reactive substances [34]; thus, total phenolic content is an adequate proxy for the phlorotannin content.

### 3.1. Antioxidant Effects

The extracts enriched with intracellular and CW-bound phlorotannins isolated from *F. vesiculosus* and *P. canaliculata* differed in their patterns of antioxidant activity. Thus, as can be seen from Table 1, the highest normalized free radical scavenging activity, discovered in the DPPH test, was observed for the intracellular phlorotannin fraction of *F. vesiculosus* (93.44%). The CW-bound phlorotannins of *F. vesiculosus* and intracellular phlorotannin-rich fraction of *P. canaliculata* yielded similar values (85.06% and 83.58%, respectively), while the extract from the cell walls of *P. canaliculata* showed a much lower activity (57.49%). On the other hand, all the tested extracts demonstrated similar electron-donating behavior, which was manifested with practically the same free cation-radical scavenging activity in the TEAC tests (about 11–14 nmol/L Trolox equivalents/μg).

Finally, the activity of the extracts in neutralizing of the superoxide anion radicals was addressed in the NBT tests. Thereby, the highest activity was detected in the extracts of CW-bound phlorotannins of both algae (49.89 nmol of O_2_^•−^/min for *F. vesiculosus* and 37.14 nmol of O_2_^•−^/min for *P. canaliculata*), whereas the efficiency of the intracellular phlorotannin-rich fractions was much lower (51.32 nmol of O_2_^•−^/min for *F. vesiculosus* and 52.04 nmol of O_2_^•−^/min for *P. canaliculata*, Table 1).

### 3.2. Antimicrobial Activity

The antibacterial activity of all four phlorotannin-enriched extracts was investigated in vitro by broth microdilution assay. Extracts were probed against several Gram-positive and Gram-negative microorganisms, and MICs were determined (Table 2). The assays revealed low or moderate antimicrobial activity for all extracts. The MIC comparison of intracellular and CW-bound phlorotannin isolates of both algae showed that, on the whole, they were quantitatively and qualitatively similar. However, the antibacterial activity of the CW extracts was generally higher in comparison to the intracellular phlorotannin-rich extracts. The highest antibacterial activities were observed against the Gram-negative strain *Escherichia coli* ATCC 25922 and the Gram-positive strain *Staphylococcus aureus* ATCC 25923 (MIC 62.5 μg/mL for intracellular phlorotannins vs. 31.2 μg/mL for CW-bound phlorotannins). The lowest activities were revealed against the Gram-positive strain MRSA ATCC 33591 (MIC 500 μg/mL for intracellular phlorotannins vs. 125 μg/mL for CW-bound phlorotannins).

Antimicrobial activities were expressed as minimal inhibitory concentrations, MICs.

### 3.3. Cytotoxic Effects of Phlorotannin-Enriched Extracts Observed with the SH-SY5Y Cell Culture

**Figure 2:**
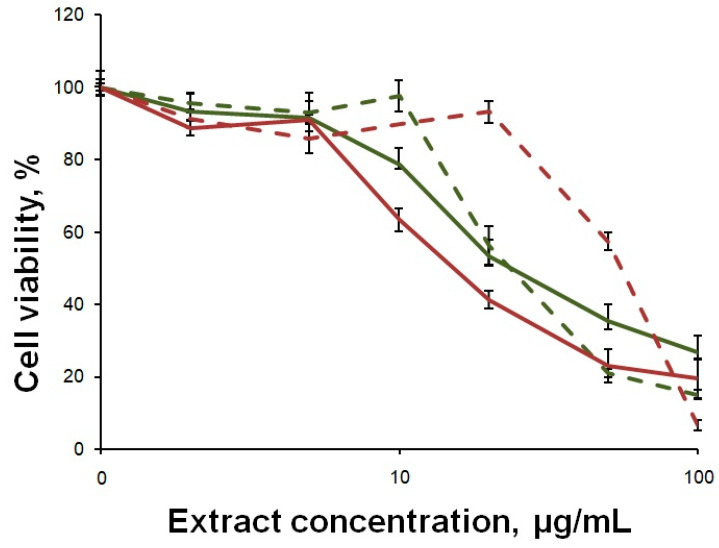
Dose–response curves (n = 4) built for cytotoxicity of the extracts of intracellular (solid lines) and CW-bound (dashed lines) phlorotannin-rich isolates obtained from *F. vesiculosus* (green lines) and *P. canaliculata* (red lines) against the differentiated culture of SH-SY5Y cells. Error bars are standard deviations; the concentration axis is scaled logarithmically.

To obtain the range of non-toxic and low-toxic concentrations of the tested algal extracts, their cytotoxicity was addressed with the differentiated SH-SY5Y cell culture prior to the assessment of the neuroprotective effects. For this, a broad concentration range (from 1 to 100 μg/mL) of the extracts was applied to the cells and to the cell-free wells as a control (to subtract the effect of the extract’s dark color from the resulting MTT signal). For all the extracts tested, the dose–effect curves were obtained, and the LD50 values could be assessed (Figure 2). In our experiments, the cell wall extracts showed slightly lower toxicity (LD50 of 24 and 54 µg/mL for *F. vesiculosus* and *P. canaliculata*, respectively) than the extracts of intracellular phlorotannins (LD50 of 25 and 16 µg/mL for *F. vesiculosus* and *P. canaliculata*, respectively). Based on these results, we selected the extract concentrations lower than 10 μg/mL for application in the further antineurodegenerative activity assay.

### 3.4. Neuroprotective (Anti-Neurodegenerative) Activity

Anti-neurodegenerative activity was assessed in two models of toxicity established for the differentiated SH-SY5Y cells. Thereby, paraquat toxicity was employed as the cellular model of Parkinson’s cell death, while the cellular model of Alzheimer’s disease relied on the Aβ25–35 toxicity.

**Figure 3:**
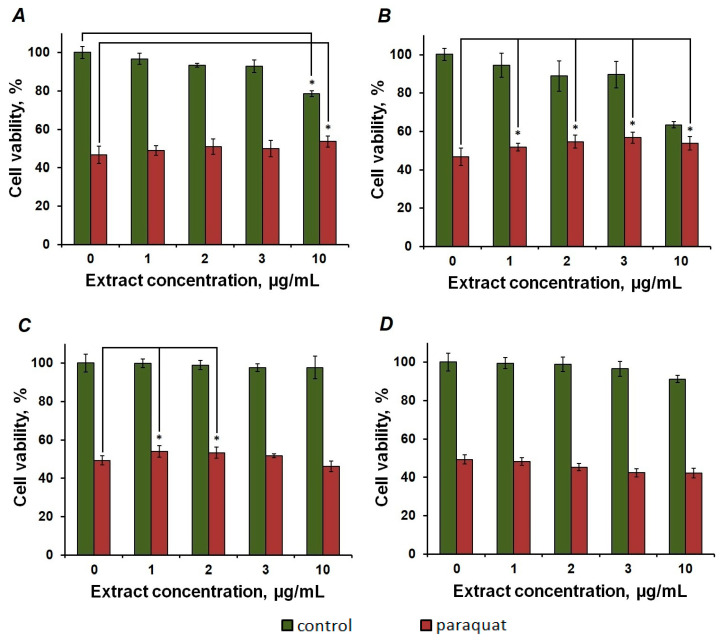
Protective effects of brown algal extracts against 800 μmol/L paraquat (PQ) toxicity evaluated by the MTT test. SH-SY5Y cells (pre-differentiated with retinoic acid and PMA) were pre-treated with intracellular phlorotannin-rich extracts of *F. vesiculosus* (**A**) and *P. canaliculata* (**B**) or extracts enriched with CW-bound phlorotannins of *F. vesiculosus* (**C**) and *P. canaliculata* (**D**) for 0.5 h, and subsequently, paraquat was applied for 24 h before the MTT application. * indicates statistically significant (*p* ≤ 0.05) effects of the treatment with the algal extracts in comparison to paraquat only. Green bars represent cells treated with various concentrations of extracts only, and red bars represent cells treated with both extracts and paraquat.

The extracts of both brown algae showed modest but significant protective effects in the paraquat cell model of Parkinson’s disease (Figure 3). Specifically, the intracellular phlorotannins of *F. vesiculosus* significantly improved the viability of paraquat-treated cells only at the highest concentration tested (10 μg/mL), which was toxic to the control cells not treated with paraquat (Figure 3A), whereas the extract of *P. canaliculata* demonstrated protective effect at all the concentrations tested (Figure 3B).

Surprisingly, although the extracts containing CW-bound phlorotannins were less toxic for the model cells, they did not demonstrate more pronounced protective effects. Thus, only the two lowest concentrations of the *F. vesiculosus* extract (1 μg/mL and 2 μg/mL) resulted in a significant increase of cell viability, whereas the cell wall extract of *P. canaliculata* thalli did not produce any protective effect (Figure 3C,D).

**Figure 4:**
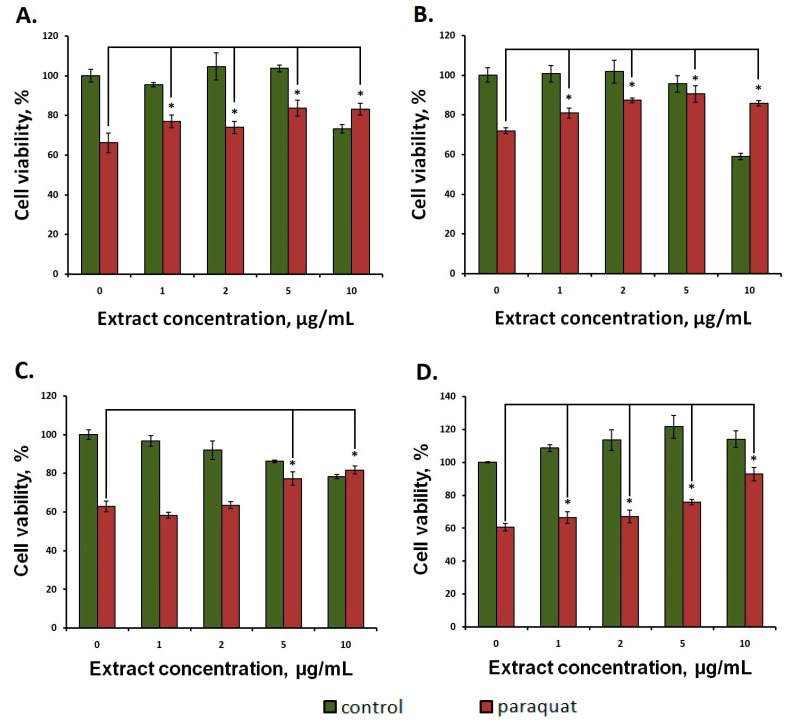
Protective effects of brown algal extracts against 25 μmol/L Aβ(25–35) toxicity evaluated in the MTT test. SH-SY5Y cells (pre-differentiated with retinoic acid and PMA) were pre-treated with the extracts rich in intracellular phlorotannins of *F. vesiculosus* (**A**) and *P. canaliculata* (**B**) or CW-bound phlorotannin extracts of *F. vesiculosus* (**C**) and *P. canaliculata* (**D**) for 0.5 h, and subsequently, Aβ(25–35) was applied for 24 h before the MTT application. * represents a statistically significant (*p* ≤ 0.05) effect of extract treatment of cells with Aβ(25–35) application in comparison to Aβ(25–35) only. Green bars represent cells treated with various concentrations of extracts only, and red bars represent cells treated with both extracts and paraquat.

In the Aβ25–35 cell model of Alzheimer’s disease, tested extracts of brown algae demonstrated well-pronounced protective activity, i.e., cell viability could be restored to almost the control levels (Figure 4). The solutions containing the extracts were applied to the cells for 0.5 h prior to the solution of Aβ(25–35), and the resulting signal was compared with the control of Aβ(25–35)-only wells. In these experiments, the extracts of *P. canaliculata* demonstrated more pronounced protective activities (practically all the concentrations tested produced observable effect) than the extracts of *F. vesiculosus* (only the highest concentrations of 5 μg/mL and 10 μg/mL were protective). The highest concentrations in the tested range produced the strongest effect, implying therapeutic doses of the extracts may be rather close to the toxic doses.

## 4. Discussion

High antioxidant potential is characteristic for most of the studied brown algae, including *F. vesiculosus* and *P. canaliculata* [35,36]. The observed ROS-protective activities of phenol-rich preparations obtained from the thalli of brown algae clearly indicate association of their pronounced antioxidant properties with the high contents of phlorotannins [37,38,39]. This observation is further supported by the high antioxidant activities of phlorotannins isolated in individual form, which were comparable or even more pronounced in comparison to raw phenol-rich isolates from the thalli of brown algae [40,41]. Thus, it was logical to assume that pronounced antioxidant activities of *F. vesiculosus* and *P. canaliculata* extracts are promising in the therapy of human diseases characterized by the high impact of oxidative stress on their pathogenesis [42,43,44,45].

As antioxidant properties can be the result different mechanisms (i.e., unspecific radical scavenging or direct transfer of electron to hydroxyl or superoxide anion radical [46,47,48]), these aspects need to be addressed. This information can be acquired by simultaneous application of several well-standardized antioxidant tests—DPPH, TEAC, and NBT assays—giving access to the total scavenging activity, direct electron transfer in redox reactions and reduction of superoxide anion [31]. As a result of the performed experiments, it was shown that for the intracellular *F. vesiculosus* and *P. canaliculata* phlorotannins, the ability to absorb free radicals was most pronounced, while the CW-bound phlorotannins of both species of brown algae showed significant activity in superoxide radical scavenging. On the other hand, all the studied isolates showed comparable activity in cation-radical neutralization. From this data, we can conclude that both intracellular and CW-bound phlorotannins have the same potential for direct cation reduction via electron donation and for the radical quenching via hydrogen atom donation. Unfortunately, the equilibrium of ROS inter-conversion is complex. Therefore, it is difficult to characterize the production and consumption of hydroxyl radicals in a direct way. However, to some extent, the antioxidant activity in respect of this ROS can be estimated by comparison of the results of DPPH and NBT assays. Exploration of Table 1 reveals similar percentage differences between activities of cell wall and cytosolic phlorotannins for both tests. This fact might indicate comparable relative contributions of the OH^•−^ scavenging activity in the antioxidant properties of both fractions.

Certainly, electron spin resonance (ESR) spectroscopy is the method of choice for analysis of radicals. However, fortunately, the diluted extracts were absolutely non-colored, and no interference with the well-established and routinely used spectrophotometric assay could be expected. Therefore, here we relied on the standard spectrophotometric test well-established in our lab [32]. Similar to the polyphenols of terrestrial plants, the phlorotannins represent reducing compounds and are readily involved in electron donation reactions. In the presence of oxidants, these reactions are accompanied by the formation of resonance-stabilized phenoxyl radicals as reaction intermediates [4,49]. Thus, the antioxidant capacity of phlorotannins should strongly depend on their potential for the formation of such phenoxyl radicals. This potential, in turn, depends of the number of phenolic moieties per molecule and their spatial orientation. It is in agreement with the well-proven fact that oligomeric compounds, especially those forming branched structures, exhibit higher antioxidant activity in comparison to low-molecular-weight representatives of the class [4,50]. Addressing several sides of the phlorotannin antioxidant activity allowed understanding the observed effects.

Generally, the high antioxidant potential of the algal extract demonstrated by our experiments is in line with previous data in the literature. However, based on the DPPH radical scavenging assay, our results clearly indicate that intracellular phlorotannin-rich extracts of *F. vesiculosus* demonstrated significantly higher activity in comparison to the isolates of *P. canaliculata* (93% vs. 84%), although the TEAC and NBT tests failed to reveal any significant differences in electron-donating properties of these preparations (Table 1). This fact might indicate higher radical scavenging potential of phlorotannins of *F. vesiculosus* in comparison to those of *P. canaliculate,* with similar electron-donating properties [34], i.e., formation of phenoxyl radicals dominated over direct reduction of ROS.

In agreement with the report of Liu et al. [19], our experiments revealed a lower antioxidant activity of the CW-bound phlorotannins in comparison to the intracellular phlorotannin fraction (both in the DPPH and NBT assays, Table 1). Thereby, for *F. vesiculosus* the difference in activity of intracellular and CW-bound phlorotannin fractions was much less pronounced than for *P. canaliculata*. For the latter, the concerted difference in electron-donating and radical-scavenging properties was observed, which might indicate uneven distribution of protective phlorotannins between the cell wall and cytoplasm. Most likely, these biochemical inter-species differences reflect ecological specialization of the algae. Indeed, in contrast to *F. vesiculosus*, which is characteristic for the low-intertidal zone, *P. canaliculata* is a high-intertidal organism that is regularly exposed to very harsh conditions, i.e., subjected to prolonged desiccation, light and temperature stress [5,51]. This definitely requires higher plasticity in metabolic adaptation, and functional differentiation of two subcellular fractions of phlorotannins may contribute to better survival of the organism.

Certainly, the observed differences in the antioxidant activity of phlorotannins need to be addressed in the context of the available information on their structure. Thus, although both *F. vesiculosus* and *P. canaliculata* have comparable distribution of oligomers by the chain length (3–39 and 3–49 phloroglucinol units, respectively), according to earlier studies, the prevalence of oligomeric compounds, including 4–12 subunits, in *F. vesiculosus* is significantly higher than that in *P. canaliculata*, while the latter predominantly accumulates higher-molecular-weight phlorotannins (Figure S1) [5,52]. It is a known fact that the antioxidant potential of phlorotannins strictly depends on the molecule structure, including the number of phloroglucinol subunits and the nature of their branching. Considering that the higher molecular weight polyphenols typically have complex structures and form multiple isomers with shielded hydroxyl groups, this may account for the lower antioxidant activity of *P. canaliculata* extracts.

Thus, in our earlier work [5], we showed that the fundamental skeleton of intracellular phlorotannins of *F. vesiculosus* and *P. canaliculata* relies mainly on aryl and ester bonds, whereas dibenzodioxin-based structures represent the only class of CW-bound phlorotannins. It can be assumed that, along with occupation of multiple hydroxyl groups for conjugation with cell wall polymers, this lower structural diversity of the CW-bound phlorotannins underlies their less pronounced antioxidant properties. On the other hand, this fact can be explained by the specificity of functions of CW-bound phlorotannins. Thus, it is known from earlier works that CW phlorotannins are incorporated into cell walls during the active growth of cells, presumably contributing to cell wall rigidity through cross-linking reactions catalyzed by vanadium-dependent haloperoxidases, and thus mainly perform a protective function against mechanical injuries and hydrodynamic burden [6]. Thus, the significantly lower antioxidant activity of the CW phlorotannins can be explained by their specific physiological properties (Table 1).

Thus, our data indicate that the CW-bound phlorotannins represent a milder antioxidant in comparison to the intra-cellular fraction. Moreover, despite the known toxicity of the CW-bound phenolics fraction [53,54], cytotoxicity of the extracts correlated with their antioxidant activity, i.e., the CW-bound phlorotannin fractions were less toxic in comparison to the intracellular ones, with the toxicity of the *P. canaliculata* the least pronounced (Figure 2). At least partly, this can be explained by the lower representation of toxic phlorotannins. Taking into account the oxidative nature of neurodegenerative processes, the CW-bound phlorotannin fractions can be assumed to be efficient neuroprotective agents. Therefore, here we addressed their anti-neurodegenerative (neuroprotective) properties in comparison to the intracellular phlorotannin-rich isolates. Thereby, two neurodegenerative amyloidogenic pathologies, namely Alzheimer’s (AD) and Parkinson’s (PD) diseases, were addressed.

Paraquat is considered as a substance inducing Parkinson’s-like pathology in animals and therefore is also used for modeling this pathology in monoaminergic cell culture (such as neuroblastoma SH-SY5Y or pheochromacytoma PC-12 [55,56,57]). Monoamine metabolism is very sensitive to oxidative stress produced by paraquat through dopamine quinone production, affecting mitochondrial function and protein folding [58,59,60]. In our experiments, we adhered to a paraquat concentration of 800 μmol/L, stably resulting in 50% differentiated SH-SY5Y cell culture viability. Activity of brown algal extracts has not been tested before in a paraquat model of neurodegeneration, but their antioxidant properties suggest the ability to prevent paraquat toxicity. The extracts demonstrated modest but significant protective effects. For *F. vesiculosus* extracts, protective activities were revealed at the highest concentration for intracellular phlorotannins and at the lowest concentrations for the fraction enriched with CW-bound phlorotannins. For *P. canaliculata*, only the intracellular phlorotannin fraction demonstrated protective activity for all concentrations tested.

Currently, using Aβ (full 1–40, 1–42 or truncated, e.g., 25–35 fragment) is considered to be a gold standard for the induction of neurotoxicity in AD research [61,62,63]. The models based on the application of Aβ appear to be easier to implement, although they suffer from several limitations. Thus, they are not able to address the progressive nature of neurodegeneration, and the concentrations of Aβ typically used for modeling are three orders of magnitude higher in comparison to the contents naturally occurring in tissues [64,65]. Among all the variants of Aβ used, the Aβ25–35 peptide has the shortest sequence (and, hence, is the easiest in synthesis and handling), but it still retains cytotoxic activity and aggregation properties of Aβ1–42 [62,63,66]. This peptide can be treated as naturally relevant, as it is present in the brains of patients with AD [66]. These characteristics make this truncated variant of Aβ one of the most widely applied models of AD-associated cytotoxicity.

Extracts of phlorotannins isolated from *F. vesiculosus* and *P. canaliculata* have already demonstrated some effectiveness in this model of AD based on the toxicity of aggregated Aβ1–42 [67,68]. Such effect can be attributed to the antioxidant properties, which were shown for phlorotannins from many brown algal species (refer to the introduction). Phlorotannins are also prone to binding proteins, which therefore can cause binding to Aβ and disaggregation, protection of cell membranes and surface receptors from interaction with toxic oligomers or enzymatic inhibition [69]. In our experiments, algal extracts demonstrated prominent protective effect against Aβ25–35 toxicity. For *F. vesiculosus,* only high concentrations produced the effect (5 and 10 μg/mL). Extracts of *P. canaliculata* revealed the activity practically at all concentrations tested, while viabilities at the highest concentrations approached the values of control untreated cells. This may be indicative of the promising neuroprotective and antineurodegenerative activity of *P. canaliculata* metabolites in the subsequent animal studies.

Antimicrobial effects represent another aspect of biological activity of phlorotannins, closely related to their characteristic structure and related antioxidant properties. Indeed, in earlier studies, it was shown that the antibacterial properties of phlorotannins are related to the ability of phenolic aromatic rings and hydroxyl groups to bind to bacterial proteins by hydrophobic and hydrophilic bonds. This process, in turn, causes inhibition of oxidative phosphorylation, changes in microbial cell permeability, and loss of internal macromolecules, which ultimately leads to the death of the microbial cell [4,70,71,72]. Taking this into account, we addressed here also the antibacterial properties of CW-bound phlorotannins. Though the MIC values observed for the intracellular phlorotannin fractions of *F. vesiculosus* and *P. canaliculata* (Table 2) were higher than those known for some other brown algae (30–60 μg/mL for phlorotannins extracted from *Ecklonia stolonifera* from the order Laminariales), they were considerably lower than the values reported for *F. spiralis* from the order Fucales (2–15 mg/mL) [73,74]. Thus, although CW-bound phlorotannin extracts showed higher antibacterial efficiency in comparison to the intracellular phenolics (the lowest MIC values 31.2 vs. 62.5 µg/mL), their antimicrobial potential is still too low to consider them as a prospective prototype for antibacterial preparations.

## 5. Conclusions

The intracellular and CW-bound phlorotannins represent clearly distinguished fractions of brown algal polyphenols featured with different bonding of phloroglucinol units in the polymeric network. Recently we identified dibenzodioxin bonding as the principal structural feature of the CW-bound phlorotannins, which differed from the intracellular fraction based mainly on the aryl and ether bonds. As the next step, we addressed the biological activities associated with the revealed structural features of the two sub-cellular phlorotannin fractions. Thereby, we compared the characteristic activity profiles of the extracts enriched in the intracellular and CW-bound phlorotannins, isolated from two fucalean algae, *F. vesiculosus* and *P. canaliculata*. This allowed efficient comparison with the available literature data that increased the validity of the results. Our study revealed several favorable biological properties of the CW-bound phlorotannin fractions of both organisms. Indeed, their lower cytotoxicity and milder antioxidant activity (in comparison to the intracellular phlorotannin-rich isolates), in combination with the neuroprotective properties, makes them promising compounds for pharmaceutical applications and the design of functional foods. However, the whole spectrum of activities needs to be This will be the next step in the study of the CW-bound phlorotannins.

## Figures and Tables

**Table 1:** The antioxidant activities assessed by DPPH, TEAC and NBT assays for the extracts enriched with intracellular and CW-bound phlorotannins of *F. vesiculosus* and *P. canaliculata*.

Extract	DPPH Normalized Activity, %	TEAC, nmol/L Trolox eq./μg	NBT Assay, nmol of O_2_^•−^/min
*Fucus vesiculosus*, CW	85.06 ± 0.04	13.82 ± 0.04	49.89 ± 0.37
*Fucus vesiculosus*, intracellular	93.44 ± 0.13	13.65 ± 0.06	51.32 ± 0.39
*Pelvetia canaliculata*, CW	57.49 ± 0.10	11.25 ± 0.03	37.14 ± 0.28
*Pelvetia canaliculata*, intracellular	83.58 ± 0.06	14.00 ± 0.03	52.04 ± 0.39
Blank	-	-	65.89 ± 0.49

DPPH—2,2-diphenyl-1-picrylhydrazyl free radical scavenging assay; TEAC—Trolox equivalent antioxidant capacity; NBT (nitroblue tetrazolium) assays—assessment of capacity to scavenge superoxide anion radicals.

**Table 2:** Antimicrobial activities of the extracts of intracellular and CW-bound phlorotannins of *F. vesiculosus* and *P. canaliculata*.

Microorganism Strain	Activity (MICs, µg/mL)			
	Intracellular Fraction		Cell Wall Fraction	
	*Fucus vesiculosus*	*Pelvetia canaliculata*	*Fucus vesiculosus*	*Pelvetia canaliculata*
*Escherichia coli* ATCC 25922	62.5	62.5	***31.2***	***31.2***
*Pseudomonas aeruginosa* ATCC 27853	250.0	125.0	125.0	62.5
*Staphylococcus aureus* SG-511	250.0	125.0	62.5	62.5
*Staphylococcus aureus* ATCC 25923	62.5	62.5	***31.2***	***31.2***
*MRSA* ATCC 33591	500.0	500.0	125.0	125.0
*Micrococcus luteus* CIP A270	62.5	62.5	62.5	62.5
*Listeria monocytogenes* EGD	250.0	250.0	125.0	125.0

## Data Availability

All data are available by request.

## Supplementary Materials

The following are available online at https://www.mdpi.com/article/10.3390/antiox12030696/s1, Figure S1: LC-MS analysis of *Fucus vesiculosus* and *Pelvetia canaliculata* phlorotannin extracts.

## Abbreviations

ABTS, 2,2′-azino-bis (3-ethylbenzothiazoline-6-sulfonic acid; AGE, advanced glycation end-product; AD, Alzheimer’s disease; BHT, butylated hydroxytoluene; CW, cell wall; DMSO, dimethyl sulfoxide; DPPH, 2,2-diphenyl-1-picrylhydrazyl; FBS, fetal bovine serum; ER, endoplasmic reticulum; HR-MS, high resolution–mass spectrometry; MHB, Mueller–Hinton broth; MIC, minimum inhibitory concentration; MTT, methylthiazolyldiphenyl-tetrazolium bromide; NADH, nicotinamide adenine dinucleotide reduced form; NBT, nitroblue tetrazolium; NMR, nuclear magnetic resonance; PBS, phosphate buffered saline; PD, Parkinson’s disease; PM, phenazine methosulfate; PMA, phorbol 12-myristate 13-acetate; ROS, reactive oxygen species; RT, room temperature; TEAC, Trolox equivalent antioxidant capacity; UPHLC-MS/MS, Ultra-performance liquid chromatography–tandem mass spectrometry.
