# Supplementary material for: First Insight into the Neuroprotective and Antibacterial Effects of Phlorotannins Isolated from the Cell Walls of Brown Algae Fucus vesiculosus and Pelvetia canaliculata
Source: Antioxidants (Basel). 2023 Mar 11;12(3):696. doi: 10.3390/antiox12030696 (PMC10045267; doi:10.3390/antiox12030696)
Supplement: Supplementary file 1 [file antioxidants-12-00696-s001.zip › antioxidants-2165407-supplementary.pdf]

The first insight in the neuroprotective and antibacterial effects of phlorotannins isolated from the cell walls of brown algae *Fucus vesiculosus* and *Pelvetia canaliculata*

Supplementary information

Darya Meshalkina,[1,2†] Elena Tsvetkova,[1,3†] Anastasia Orlova,[4] Renata Islamova,[1] Maria Grashina,[1] Daria Gorbach,[1] Vladimir Babakov,[5] Antonio Francioso,[6,7] Claudia Birkemeyer,[8,9] Luciana Mosca,[7] Elena Tarakhovskaya,[1,10*] Andrej Frolov[4*]

[1]St. Petersburg State University, St. Petersburg, Russia; [2]Sechenov Institute of Evolutional Physiology and Biochemistry; [3]Institute of Experimental Medicine, Department of General Pathology and Pathological Physiology, St. Petersburg, Russia; [4]K.A. Timiryazev Institute of Plant Physiology RAS, Laboratory of Cell Regulation, Moscow, Russia; [5]Research Institute of Hygiene, Occupational Pathology, and Human Ecology, Federal Medicobiological Agency, St. Petersburg, Russia; [6]Sapienza University, Department of Biochemical Sciences, Roma, Italy; [7]Agriculture and Environment University of Teramo, Department of Bioscience and Technology for Food, Teramo, Italy; [8]University of Leipzig, Faculty of Chemistry and Mineralogy, Leipzig, Germany; [9]German Center for Integrative Biodiversity Research (iDiv), Leipzig, Germany; [10]Vavilov Institute of General Genetics RAS, St. Petersburg Branch, St. Petersburg, Russia

[†]These authors contributed equally to the manuscript

*Corresponding authors:

Dr. Andrej Frolov
K.A. Timiryazev Institute of Plant Physiology RAS
Laboratory of Analytical Biochemistry and Biotechnology
Botanicheskaya Ulitsa, 35, Moscow
127276, Moscow, Russian Federation
Tel. +7(499) 678-54-00,
E-mail: frolov@ifr.moscow

Dr. Elena Tarakhovskaya
St. Petersburg State University
Department of Plant Physiology and Biochemistry
Universitetskaya nab. 7-9,
199034, St. Petersburg, Russia
Tel. +78123289695/Fax. +78123289703
Email: elena.tarakhovskaya@gmail.com

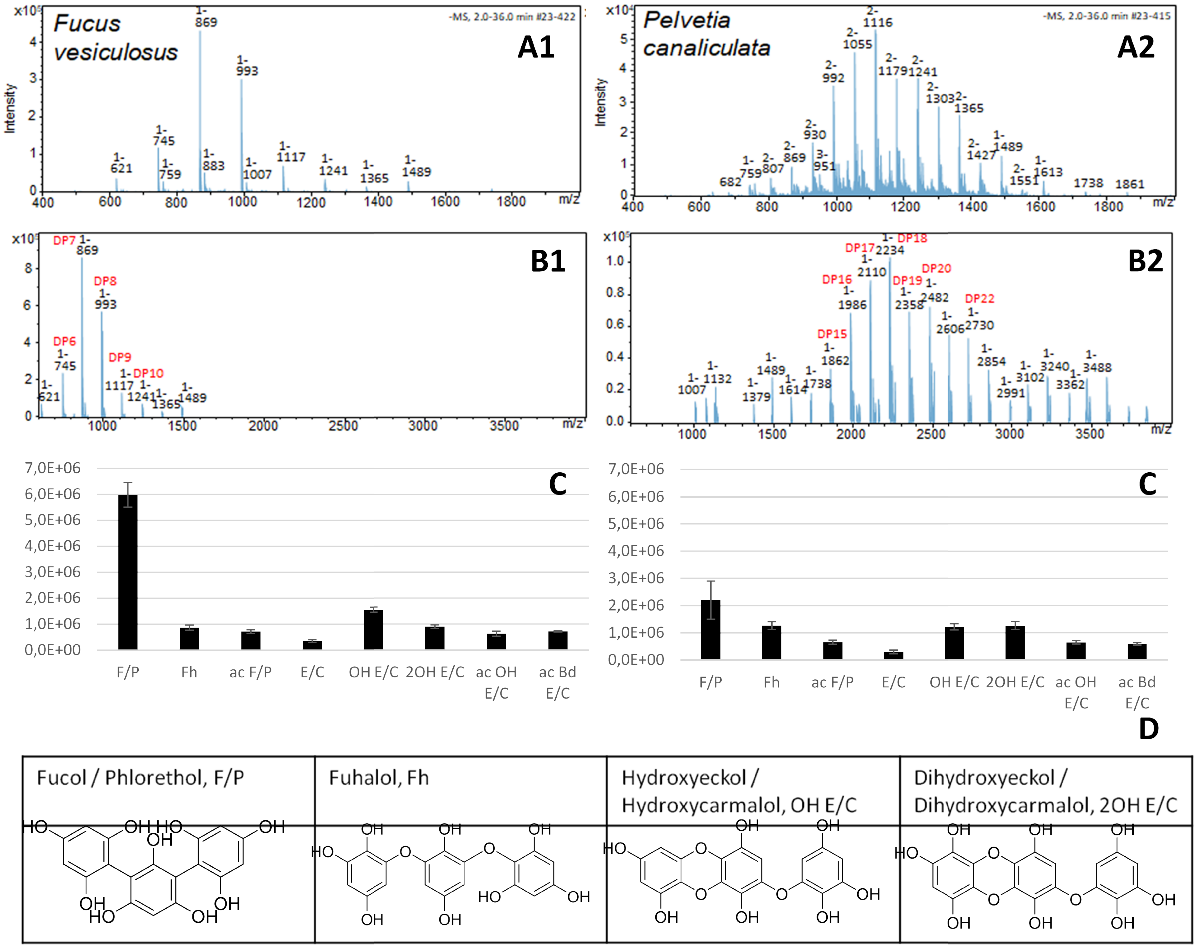

Figure S1 LC-MS analysis of *Fucus vesiculosus* and *Pelvetia canaliculata* phlorotannin extracts. Graphs and spectra were relay on the data published by Birkemeyer et al. (2020). Information on materials and methods can also be found there. **A.** The **s**pectra averaged over the chromatogram region in which phlorotannins elute (**A1** and **A2** for *F. vesiculosus* and *P. canaliculata*, respectively). These spectra show the most abundant phlorotannin species in the corresponding extracts. **B.** Charge-deconvoluted spectra (**B1** and **B2** for *F. vesiculosus* and *P. canaliculata*, respectively) show the masses of the present phlorotannin species and the relative intensities of their signals. The most abundant species are labeled by their corresponding degree of polymerization (DP). **C.** Quantitative distribution of the most abundant phlorotannin series identified in the extracts of *F. vesiculosis* (**C1**) and *P. canaliculata* (**C2**): F/P - fucols/phlorethols, Fh - fuhalols, ac F/P - acetylated fucols/phlorethols, E/C - eckols/carmalols, OH E/C - hydroxylated eckols/carmalols, 2OH E/C - dihydroxylated eckols/carmalols, ac OH E/C - acetylated hydroxyl eckols/carmalols, ac Bd E/C - acetylated benzodioxin-eckols/carmalols. **D.** Principal structure of the four most abundant phlorotannin series at DP 3. Further details and the structural skeleton of other phlorotannin series identified in the extracts can be found in Birkemeyer et al. 2020 (Birkemeyer, C.; Lemesheva, V.; Billig, S.; Tarakhovskaya, E. Composition of intracellular and cell wall-bound phlorotannin fractions in fucoid algae indicates specific functions of these metabolites dependent on the chemical structure. Metabolites. 2020, 10, 369).
